# Localised ABA signalling mediates root growth plasticity

**DOI:** 10.1016/j.tplants.2013.08.009

**Published:** 2013-10

**Authors:** Zhaojun Ding, Ive De Smet

**Affiliations:** 1 The Key Laboratory of Plant Cell Engineering and Germplasm Innovation, Ministry of Education, School of Life Sciences, Shandong University, Jinan, Shandong 250100, PR China; 2 Department of Plant Systems Biology, VIB, Technologiepark 927, B-9052 Ghent, Belgium; 3 Department of Plant Biotechnology and Bioinformatics, Ghent University, Technologiepark 927, B-9052 Ghent, Belgium; 4 Division of Plant and Crop Sciences, School of Biosciences, University of Nottingham, Sutton Bonington Campus, Loughborough, Leicestershire LE12 5RD, UK

**Keywords:** abscisic acid, primary root growth, lateral root development, endodermis

## Abstract

Two recent reports show that cellular abscisic acid (ABA) signalling, together with other phytohormone signalling pathways, is crucial for salt-regulated root growth dynamics. Here we discuss these findings and place them in a broader framework on how cellular hormone signalling regulates root growth plasticity in response to environmental cues.

Root system architecture supports plant growth and allows plants to acclimate to ever changing environmental growth conditions. Environmental cues such as water and nutrient availability, salt, gravity and light have profound effects on plant root system architecture. Plant hormones are important secondary signalling molecules that mediate responses of root systems to such environmental stimuli [1]. Intriguingly – tissue or cell specific signalling networks are essential for plant hormone-regulated root growth and development. For example, brassinosteroid (BR) and gibberellic acid (GA) signalling, in the epidermis and endodermis, respectively, regulate root meristem size [2]. However, how environmental stimuli are translated into tissue or cell specific signalling through the action of plant hormone-triggered root growth dynamics is not fully understood.

ABA, which is known to be involved in stress responses, has been found to play a key role in lateral root formation 3, 4. Recently, two reports from the Dinneny group 5, 6 have unveiled how ABA signals are transduced in a cell specific manner and integrated with other plant hormones, thereby regulating lateral root development in response to saline environments.

Duan *et al.* [5] found that primary and lateral roots have intrinsically different response programs to salinity, with lateral roots having a stronger suppression than primary roots after salt treatment (Figure 1). To test whether salt stress-induced root system architecture changes are mediated through ABA, which is important for root development, the authors measured the effect of salt on root growth for various mutants disrupted in the ABA signalling pathway. Their analysis indicated that mutants that have defects in ABA biosynthesis, signal transduction, and ABA-regulated gene transcription exhibited strongly reduced salt-suppressed lateral root growth [5]. In addition, after salt treatment, the lateral root-specific higher expression of the ABA-responsive *ProRAB18::GFP* reporter further suggested that the differential response of primary and lateral roots to salt treatment is due to the differences in ABA signalling. This indicated that ABA signalling is crucial for salt stress-induced lateral root suppression. By contrast, the primary root has unaffected sensitivity to salt treatment in ABA-related mutants.

**Figure 1:**
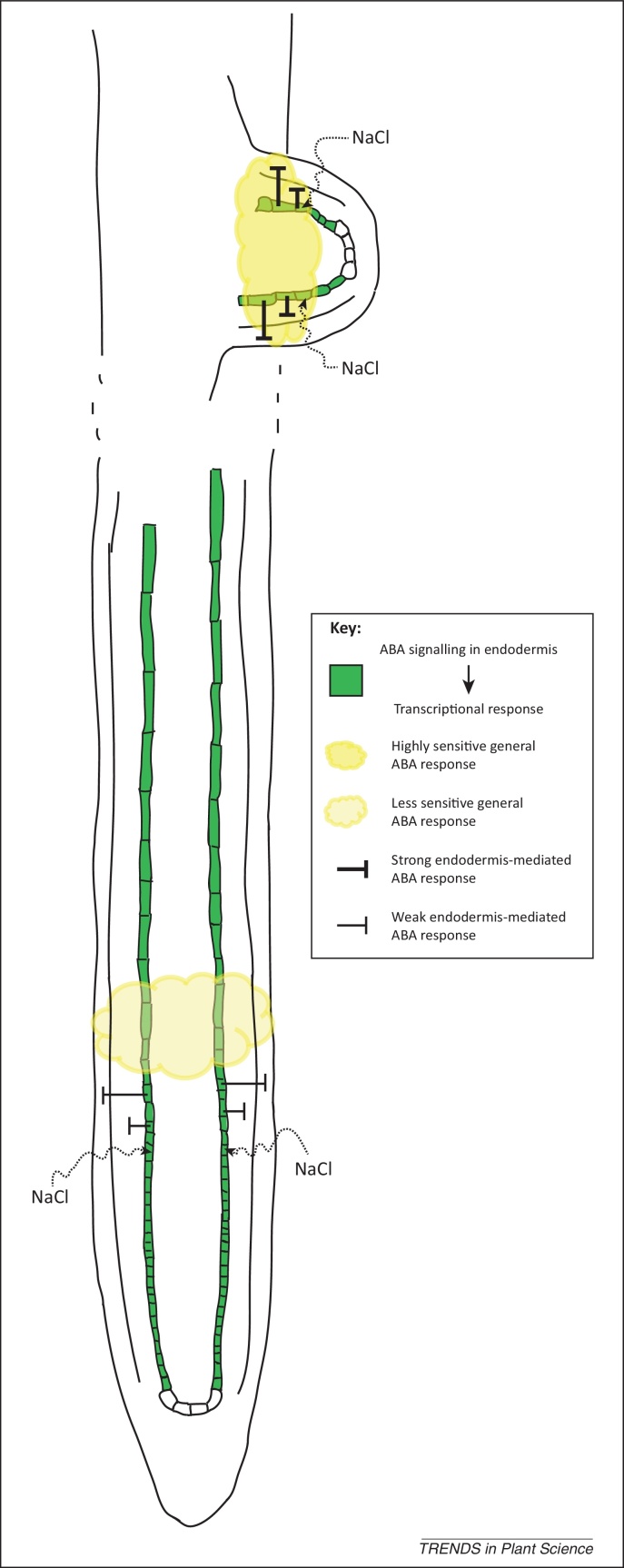
Salt (NaCl)-mediated regulation of root system architecture via endodermis-specific abscisic acid (ABA) response. Green, endodermis.

## ABA signalling in endodermis regulates root growth under saline conditions

Cell specific nitrogen responses mediate root growth plasticity, which is largely through nitrogen-induced AUXIN RESPONSE FACTOR 8 (ARF8) in the pericycle [7]. This indicates that tissue or cell specific plant hormone signalling is probably crucial for mediating environmental stimuli and therefore root growth plasticity. The recent reports from the Dinneny group further support the above scenario 5, 6.

Duan *et al.* [5] used the GAL4-VP16/UAS enhancer trap system to drive tissue-specific expression of *abi1-1*, which dominantly suppresses ABA signalling. They found that *abi1-1* was most effective at rescuing lateral root growth during salt treatment when expressed in the endodermis, and less so when expressed in the epidermis or cortex. Consistent with the genetic data of lateral root specificity of ABA response, expression of *abi1-1* in the various tissue layers, including endodermis, had little effect on primary root growth in the presence of 100 mM NaCl [5].

Using live-imaging analysis, Geng *et al.* [6] observed that salt-regulated root growth is dynamically regulated with a period of quiescence followed by recovery and then homeostasis. Live-imaging analysis further revealed that endodermis-specific activation of *abi1-1* delayed root growth recovery and reduced growth during the homeostasis [6]. Transcript profiling on root tips expressing *abi1-1* in the endodermis revealed that many salt-regulated genes showed a correlation between their expression pattern based on the spatio-temporal map and the endodermis where ABA signalling was most critical for salt regulation [6].

Both studies indicate that ABA signalling in the endodermis is essential for salt-regulated lateral root growth dynamics via regulating spatially localised transcriptional programs. ABA signalling plays, however, minor roles in salt-regulated primary root growth repression because the same concentration of salt induced less ABA signalling (Figure 1).

## Cellular hormone crosstalk during salt-regulated root growth dynamics

Highly dynamic spatio-temporal ABA signalling is involved in salt-regulated root growth dynamics. To figure out if other plant hormones are involved in ABA-mediated salt stress-regulated lateral root growth changes, Duan *et al.* [5] also investigated the roles of GA in this process. Through mutant analysis, chemical treatments and cellular analysis of *REPRESSOR OF GA* (*RGA*) expression, ABA and GA signalling are found to act in opposing ways to regulate lateral root growth and this regulation occurs through mutual antagonism as well as independent pathways.

Using fluorescence-activated cell sorting of GFP-marked cell types, Geng *et al.* [6] generated a spatio-temporal transcriptional map of the salt stress response that spanned the various phases of growth they observed through live imaging in specific cell or tissue types. They found that plant hormone and salt response clusters showed significant overlap, indicating that plant hormones are important intermediary signals regulating growth downstream of salt treatment. The spatio-temporal transcriptional map showed that ABA, jasmonic acid (JA), BR, and GA signalling are associated with the largest number of salt-responsive clusters. Interestingly, GA and BR targeted the same set of salt-regulated gene clusters and the dynamic repression of both GA and BR signalling during salt stress regulates primary root growth quiescence. Similar to GA signalling, BR signalling – shown through the *ProBZR1:BZR1:YFP* reporter – was temporarily suppressed during the early phase of the salt response and recovered later. This is important for promoting growth in the recovery stage of salt stress. It remains to be seen whether ABA and BR signalling also act in opposing ways to regulate lateral root growth.

## Root growth dynamics in response to other environmental cues

The two recent reports from the Dinneny group are good examples to show how ABA signalling mediates salt stress-regulated root growth dynamics 5, 6. Besides salt stress, light, gravity, and nutrients, such as nitrate and phosphate, are also important cues that are continuously affecting plants growth and that can impact on root system architecture. In the past few years, the roles of plant hormones in mediating environmental cue-regulated root growth have started to emerge. ABA was reported to be involved in hydrotropic response in roots of *Arabidopsis thaliana* through crosstalk with light signalling [8]. Auxin, which has long been known to mediate lateral root initiation and root gravitropic response, was identified as a long-distance signal in response to light and shown to be involved in light-regulated root growth changes [9]. Nitrate was found to modify root system architecture through ABA-induced lateral root primordium arrest [10] and through cell specific nitrogen responses intersecting with auxin response in the pericycle [7]. All these investigations clearly show that environmental stimuli regulate root growth plasticity largely through plant cellular hormone signalling.

## Concluding remarks and open questions

Collectively, the two recent studies 5, 6 not only make an important contribution to our understanding of how cellular ABA signalling is involved in salt stress-regulated root growth dynamics (Figure 1) but they also provide a very good example to illustrate how plant cellular hormone signalling regulates root growth plasticity in response to environmental cues. However, there are still some open questions regarding the crosstalk between ABA and other plant hormones, such as GA and BR, during salt stress-regulated root growth dynamics. Although ABA-regulated root growth dynamics largely relies on ABA signalling in the endodermis, it is still uncertain whether GA and BR regulated root growth in response to salt stress is also dependent on their signalling in the endodermis. It will be interesting to investigate if salt stress can influence GA accumulation in the endodermis and whether this requires ABA signalling in the endodermis.
